# Observation of the protein expression level *via* naked eye: Pt clusters catalyze non-color molecules into brown-colored molecules in cells

**DOI:** 10.3389/fchem.2023.1145415

**Published:** 2023-02-13

**Authors:** Dongfang Xia, Yong Zhang, Chunyu Zhang, Xiuxiu Yao, Yuhua Tang, Fuchao Wang, Xu Han, Hongzong Yin, Chao Xu, Xueyun Gao

**Affiliations:** ^1^ College of Chemistry and Material Science, Shandong Agricultural University, Taian, Shandong, China; ^2^ Faculty of Environment and Life, Beijing University of Technology, Beijing, China; ^3^ Institute of Materia Medica, Shandong First Medical University & Shandong Academy of Medical Sciences, Jinan, Shandong, China; ^4^ CAS Key Laboratory for Biomedical Effects of Nanomaterials and Nanosafety, Multi-disciplinary Research Division, Institute of High Energy Physics and University of Chinese Academy of Sciences (UCAS), Chinese Academy of Sciences (CAS), Beijing, China

**Keywords:** Pt cluster, *α*_*v*_ *β*_*3*_, peroxidase-like catalysis, tumor cells, diaminobenzidine

## Abstract

*α*_v_ *β*_3_ is overexpressed in various tumor cells and plays a key role in tumor genesis, invasion, and metastasis. Therefore, it is of great significance to precisely detect the *α*_v_ *β*_3_ level in cells via a simple method. For this purpose, we have constructed a peptide-coated platinum (Pt) cluster. Due to its bright fluorescence, well-defined Pt atom numbers, and peroxidase-like catalytic activity, this cluster can be used to evaluate *α*_v_ *β*_3_ levels in cells by fluorescence imaging, inductively coupled plasma mass spectrometry (ICP-MS), and catalytic amplification of visual dyes, respectively. In this report, the expression level of *α*_v_ *β*_3_ in living cells is well-detected by the naked eye under an ordinary light microscope when the Pt cluster binds to αvβ3 in cells and catalyzes non-color 3,3′-diaminobenzidine (DAB) into brown-colored molecules *in situ*. Moreover, SiHa, HeLa, and 16HBE cell lines with different *α*_v_ *β*_3_ expression levels can be visually distinguished by the peroxidase-like Pt clusters. This research will provide a reliable method for the simple detection of *α*_v_ *β*_3_ levels in cells.

## 1 Introduction

Integrins are a superfamily of cell adhesion receptors that can bind to extracellular matrix ligands, surface ligands, artificial ligands, and the transmembrane *αβ* heterodimers with at least 18 *α* and 8 *β* subunits and are capable of forming at least 24 different heterodimers (Takada et al., 2007; Marelli et al., 2013; Chen et al., 2016; Demircioglu and Hodivala-Dilke, 2016; Reis et al., 2022). Among them, *α*_v_ *β*_3_ can mediate adhesion, malignant transformation, tumor growth, invasion, and metastasis at different stages of cancer (Knowles et al., 2013; Tang et al., 2020) and is involved in the regulation of angiogenesis (Danhier et al., 2012; Echavidre et al., 2022). *α*_v_ *β*_3_ is highly expressed in tumors rather than normal tissues (Pu et al., 2019; Nakagawa et al., 2022), and its expression is associated with the disease progression of various tumor types (Lin et al., 2018; Cheng et al., 2021). Therefore, there is an urgent need to develop an efficient method for the detection of *α*_v_ *β*_3_ in cells.

At present, the conventional detection methods of protein expression are Western blotting (Aksamitiene et al., 2007; Schnell et al., 2008), enzyme-linked immunoassay (ELISA) (Rissin et al., 2010; Radwan et al., 2019), and immunofluorescence (Zinchuk et al., 2007; Seraya-Bareket et al., 2020). These methods are capable of well-evaluating the cellular protein expression level. However, the requirement of cell lysis and protein extraction in the pretreatment of immunoblotting or ELISA makes them unsuitable for the detection of proteins in intact cells. Immunofluorescence can realize *in situ* evaluation of protein expression in cells, but the fluorescence of fluorophore-labeled antibodies is easy to be quenched. In addition, there are many background fluorescence signals in biological samples, thereby leading to false results. Therefore, it is necessary to develop a simple and accurate detection method for *in situ α*_v_ *β*_3_ detection in cells.

Metal clusters such as gold or copper nanoclusters possess good optical properties and precise molecular composition. Meanwhile, the unique biological effects and catalytic properties of metal ions make metal clusters good candidates for biomedical imaging and tumor therapy. In recent years, different polypeptide functional metal cluster biomarkers were employed to label and quantitatively analyze proteins on the cell surfaces. In principle, the surface protein analysis takes advantage of the functional peptide affinity properties similar to an antibody with antigens and the precise molecular composition of clusters. Meanwhile, the excellent optical properties and catalytic activity can eventually realize the fluorescence imaging and catalytic color imaging of the labeled proteins. For example, a peptide with the specific sequence of H_2_N-CCYKKKKKKLFSHAVSSNG-COOH was designed to synthesize Au clusters. The fragment LFSHAVSSNG specifically targets E-cadherin on the surface of lung tumor cells. Cells overexpressing E-cadherin were detected to have more Au clusters (Li et al., 2022). Au-CCYHWKHLHNTKTFL clusters had been constructed to specifically detect and quantify the membrane-anchored membrane type-1 matrix metalloproteinase (MT1-MMP) overexpressed on cancer cells (Zhang et al., 2018). Recently, a high-affinity PSMA-targeting ligand (PSMA-1) was developed, and it demonstrated its use in prostate cancer imaging and photodynamic therapy. The PSMA-1 ligand is further modified for *in situ* synthesis of PSMA-targeted Au25 clusters (CY-PSMA-1-Au25), providing a high-affinity and highly effective radiosensitizer for prostate cancer (Luo et al., 2012).

**FIGURE 1:**
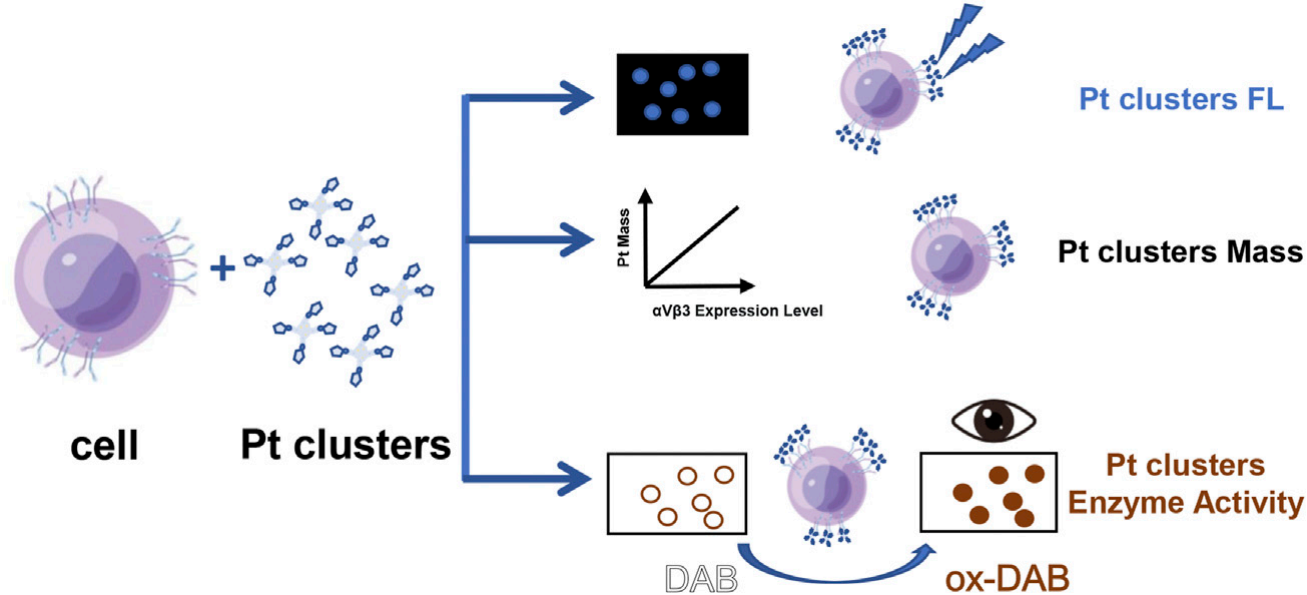
Schematic illustration of the method to detect integrin in cells *via* Pt clusters.

In this study, Pt clusters were synthesized using Cyclo (-RGDfK)-YCC, a peptide specifically targeting *α*_v_ *β*_3_, as a template. Due to the well-defined molecular structure, the as-synthesized peptide-coated Pt clusters show blue fluorescence and horseradish peroxidase activity. The Pt clusters can catalyze the oxidation of non-color 3,3′,5,5′-tetramethylbenzidine (TMB) into blue color and catalyze 3,3′-diaminobenzidine (DAB) into brown-colored molecules, respectively. In addition, the peptide-coated Pt clusters can specifically target *αvβ3* on tumor cell membranes. When the Pt clusters bind to *α*_v_ *β*_3_ on the cell membrane, DAB is catalyzed and generates brown color on the cell membrane, which can be observed by ordinary optical microscopy. Three cell lines with different expression levels of *αvβ3* were detected: SiHa (high expression), HeLa (low expression), and 16HBE (no expression). Different brown color intensities of these cell lines were observed under ordinary light microscopy. In addition, the accuracy of the method was verified by laser confocal microscopy and inductively coupled plasma mass spectrometry (see illustration in Figure 1).

## 2 Materials and methods

### 2.1 Materials

H_2_PtCl_6_ was purchased from the Shanghai Fine Chemical Materials Research Institute (AR, Shanghai, China). The peptide cyclo-(RGDfK)YCC was purchased from Beijing SciLight Biotechnology Co., Ltd. Phosphate buffer solution (PBS) and trypsin-EDTA were obtained from Gibco (NY, United States of America). Hydrogen peroxide (H_2_O_2_), sodium hydroxide (NaOH), nitric acid (HNO_3_), and hydrochloric acid (HCl) were obtained from Beijing Chemical Reagent Co., Ltd. 3,3′,5,5′-Tetramethylbenzidine (TMB), DAB horseradish peroxidase color development kit, and YO-PRO-1/RNase staining solution for nucleus were obtained from Beyotime Biotech. Inc. Ultrapure water (18.2 MΩ) was used throughout the experiments.

### 2.2 Synthesis of the Pt cluster

Briefly, the H_2_PtCl_6_ (50 mM, 200 μL) solution was added to cyclo-(RGDfK)YCC (0.5 mM, 5 mL) solution under vigorous stirring at 42°C. Subsequently, the NaOH (1 M, 100 μL) solution was injected into the reaction system. After 12 h of reaction under dark conditions and gentle stirring, the cluster was purified and concentrated using an ultrafiltration tube (molecular weight cutoff: 3 kDa) to remove free peptides and ions.

### 2.3 Characterization of Pt clusters

The optical properties of Pt clusters were characterized using a spectrofluorometer (RF-5301, Shimadzu, Japan) and a UV-1800 spectrophotometer (Shimadzu, Japan). The Pt cluster sizes and zeta potential were recorded on a Zetasizer Nano (ZS90, UK) instrument. The molecular formula of Pt clusters was analyzed by matrix-assisted laser desorption/ionization time-of-flight mass spectrometry (MALDI-TOF MS, ABI MALDI-TOF, United States) in the positive ion linear mode by using sinapinic acid as the matrix. The TEM image was obtained using an FEI Titan G2 (300 kV) transmission electron microscope.

### 2.4 ICP-MS analysis of the Pt concentration in cells

After Pt clusters were bound to the integrin of the cell, free Pt clusters were washed out, and the integrin-bound Pt was measured using an inductively coupled plasma mass spectrometry (ICP-MS) analysis system (Thermo Elemental X7, United States). The cell was added to aqua regia (HCl: HNO_3_ = 3:1) overnight, and then, the mixture was heated to 160°C. After the solution was concentrated to 10 mL, it was diluted with an aqueous solution containing 2% HNO_3_ and 1% HCl to a final volume of 10 mL. All samples were injected into the ICP-MS system in turn, and each sample was measured three times to obtain an average value. Bismuth (20 ppb, containing 2% HNO_3_ and 1% HCl) solution was used as the internal standard solution. By testing a series of Pt standard aqueous solutions (0.1, 0.5, 1, 5, 10, and 50 ng mL^−1^), a standard plot of Pt standard concentration was obtained.

### 2.5 Peroxidase-like activity of Pt clusters

After Pt clusters catalyzed TMB in solution, the non-color TMB produced a soluble blue-colored TMB with absorbance at 652 nm. To verify the peroxidase-like activity of Pt clusters, Pt cluster solution (0, 20, 40, 80, 120, and 160 μM Pt) with 100 mM H_2_O_2_ and 800 µM TMB was incubated under neutral conditions for 5 min. Then, the color of the reaction solutions was monitored, and the absorbance at 652 nm was measured using a SpectraMax M2 microplate reader.

### 2.6 Pt cluster specifically binding to the integrin on cell membranes

Human cervical carcinoma cell lines (SiHa and HeLa) and human bronchial epithelial cell lines (16HBE) were purchased from the Cancer Institute and Hospital, Chinese Academy of Medical Sciences. Cells were cultured in RPMI 1640 (HyClone, United States) supplemented with 10% fetal bovine serum (FBS) and 1% penicillin–streptomycin (Gibco, United States). Three kinds of cells (2 × 10^4^ cells) were seeded and plated in a glass-bottom dish and incubated at 37°C for 24 h. After removing the medium, the cells were washed with PBS and fixed with 4% paraformaldehyde for 30 min. Then, 3% BSA was added and incubated for 1 h to block the non-specific recognition sites. The cells were then incubated with Pt clusters for 1 h. Untreated cells were used as control. Then, the nucleus was stained with YO-PRO-1/RNase Staining Solution (1 mL) for 10 min. Finally, the fluorescence images of cells were captured using a Nikon Ti-e microscope with a 60 × 1.4NA plan apochromatic oil immersion lens.

To further confirm the specificity of Pt clusters binding to *α*_v_ *β*_3_, we designed a competitive and blocking binding site study. In the competitive binding site study, the cells were incubated with PBS containing 5 mM RGD peptide and 120 μM Pt clusters for 1 h. The nucleus was then labeled with YO-PRO-1/RNase Staining Solution for 5 min. After washing with PBS three times, the cells were used for cell fluorescence imaging. In the blocking binding site study, the cells were incubated with 5 mM RGD (1 mL) solution for 1 h, washed with PBS, and then incubated with Pt cluster (120 μM, 1 mL) solution for another 1 h. The cells were then washed with PBS, and the nucleus was stained with YO-PRO-1/RNase Staining Solution. Finally, the cells were used for cell fluorescence imaging.

### 2.7 Pt cluster concentration optimization in cells

SiHa cells were fixed with 4% paraformaldehyde for 30 min, sealed with 3% BSA, washed with PBS, and incubated with Pt clusters of different Pt concentrations (0, 40, 80, 120, 160, and 200 μM) for 1 h. After imaging was completed, the DAB working solution was added and incubated at 37°C for 30 min. The cells were thoroughly washed with PBS, and cells dyed with brown DAB were observed using an ordinary optical microscope.

### 2.8 Comparison of fluorescence and DAB imaging of SiHa, HeLa, and 16HBE cells

SiHa, HeLa, and 16HBE cells with different *α*_v_ *β*_3_ expressions were fixed with 4% paraformaldehyde, and the non-specific binding sites of fixed cells, with 3% BSA. The cells were incubated with 120 μM Pt clusters for 1 h and then washed with PBS, and fluorescence imaging was performed with the CLSM system. For all three cell lines, the DAB working solution was added and incubated at 37°C for 30 min. After cleaning with PBS, they were observed under an ordinary light microscope.

### 2.9 Analysis of the *αvβ3* expression level *via* ICP-MS

The non-specific binding sites of fixed cells were first blocked with 3% BSA. These fixed cells were incubated with 120 μM Pt clusters for 1 h to specifically label. The sample was transferred to a beaker and pre-digested with 3 mL nitric acid and 1 mL hydrogen peroxide for 12 h. After being concentrated to 0.1 mL at 160°C, the mixture was digested with aqua regia for another 12 h and then was concentrated again to 0.1 mL. Finally, an aqueous solution containing 2% HNO_3_ and 1% HCl was used to dilute the concentrated solution to an appropriate volume. The Pt content in the diluted solution was determined by ICP-MS.

### 2.10 Statistical data analysis

Fluorescence intensity analysis: in brief, a blue fluorescence image was opened, converted to 8-bit, inverted, uncalibrated OD, scale was set, measurements and threshold were set, and measured. DAB intensity analysis: by measuring the gray value, DAB intensity was reflected, a DAB image was opened, converted to 8-bit, uncalibrated OD, measurement and threshold were set, and measured. All data are represented as the mean ± SD. Statistical comparisons were conducted using one-way analysis of variance (ANOVA). *p < 0.1, **p < 0.05, and ***p < 0.001 were defined as statistically significant difference.

## 3 Results and discussion

### 3.1 Preparation and characterization of Pt clusters

**FIGURE 2:**
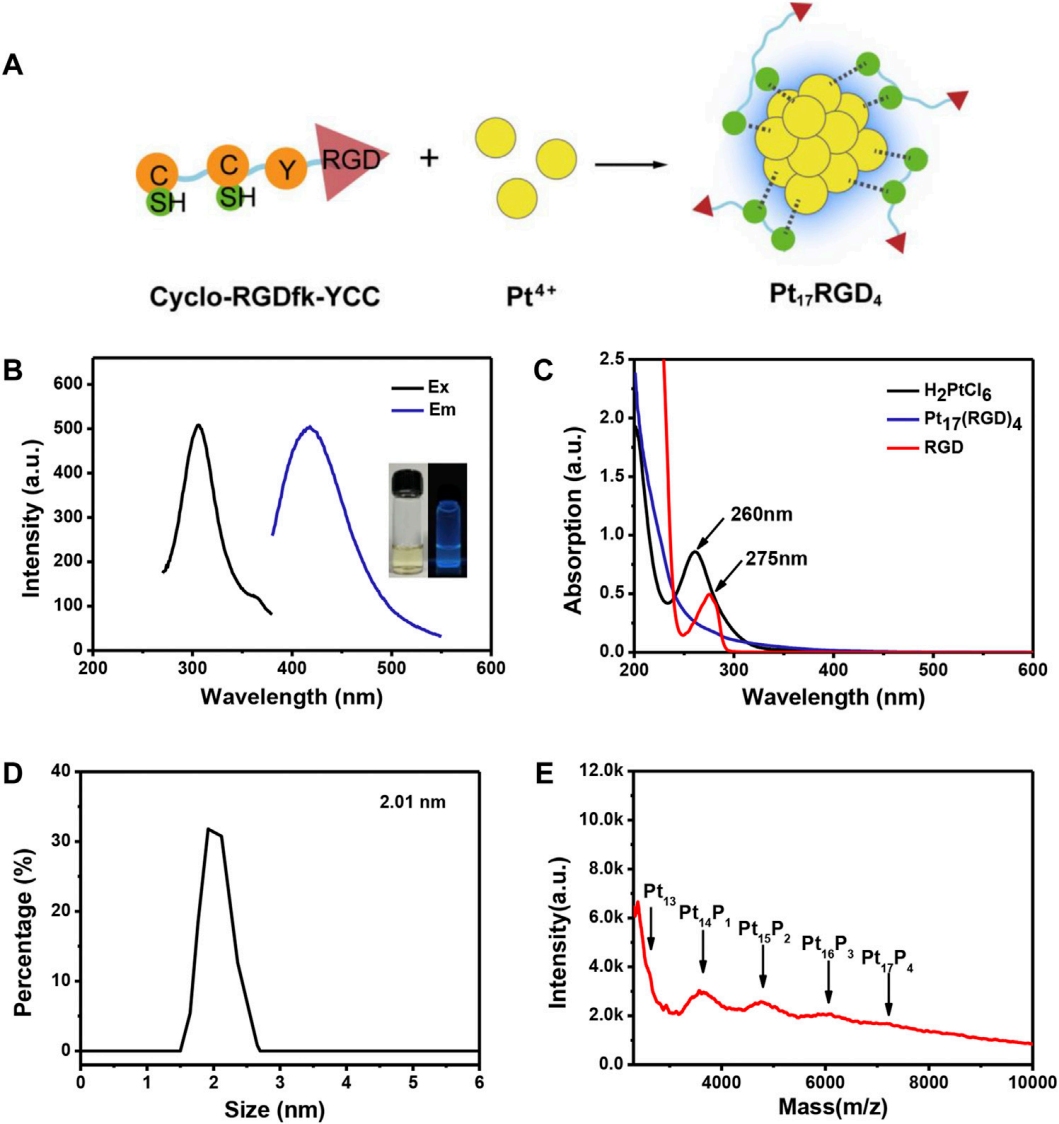
Characterization of Pt clusters. **(A)** Schematic diagram of the synthetic process of Pt clusters. **(B)** Fluorescence excitation (black line, 306 nm) and emission (blue line, 418 nm) spectra of the Pt clusters; the insets present images of the product under visible (left) and 365-nm portable UV light (right).**(C)** UV-Vis spectra of the RGD peptide (red line), H_2_PtCl_6_ (black line), and Pt clusters (blue line). **(D)** Size distribution of the Pt clusters obtained from dynamic light scattering. **(E)** MALDI-TOF-MS analysis of Pt clusters.

Figure 2A illustrates the synthesis of Pt clusters, the cyclo-(RGDfK)YCC peptide that contains two functional domains, domain cyclo-(RGDfK) is a specific sequence of targeted *αvβ3* (Nieberler et al., 2017), and CCY can reduce Pt^4+^ to Pt atoms and bind Pt atoms *via* sulfhydryl groups of cysteine (Zhang et al., 2020). The peptide-coated Pt clusters were further purified and used for a follow-up study. The fluorescence characteristics of the Pt clusters were measured by fluorescence spectrophotometry. The maximum excitation peak of the Pt cluster was 306 nm (Figure 2B, black curve), and the maximum emission peak was 418 nm (Figure 2B, blue curve). The Pt cluster fluorescence was highly stable at 4°C in the system of H_2_O with no significant changes for over 18 days (Supplementary Figure S1). The transparent Pt cluster solution appears pale yellow under natural light and fluorescently blue under UV light (Figure 2B). In addition, the formation of Pt clusters was determined by ultraviolet visible spectrophotometry. Pt^4+^ in aqueous solution had a strong absorption at 260 nm (Figure 2C, black line), and the absorption peak of the peptide was at 275 nm (Figure 2C, red line), which was attributed to the π–π conjugate structure of the phenol side group in tyrosine (Zhang et al., 2021; Zhang et al., 2022). There was no obvious absorption peak after the formation of Pt clusters. Dynamic light scattering (DLS) measurements show that the Pt cluster has a hydrodynamic diameter of about 2.01 nm (Figure 2D). A typical transmission electron microscopic (TEM) image is presented in Supplementary Figure S2. Furthermore, the precise composition of the Pt cluster was characterized by matrix-assisted laser desorption/ionization time-of-flight mass spectrometry (MALDI-TOF-MS) (Luo et al., 2012; Du et al., 2022). Figure 2E illustrates a series of strong peaks between 2,500 and 10,000 m/z in the mass spectrum. The mass spectra are composed of serial main peaks with a space of about 1168 m/z between adjacent peaks, which matches the molecular weight of one Pt atom and a peptide. The strongest main peak was located at around 7,207 m/z, which matches the peak of Pt_17_ (RGD)_4_.

### 3.2 Catalytic property of Pt clusters

**FIGURE 3:**
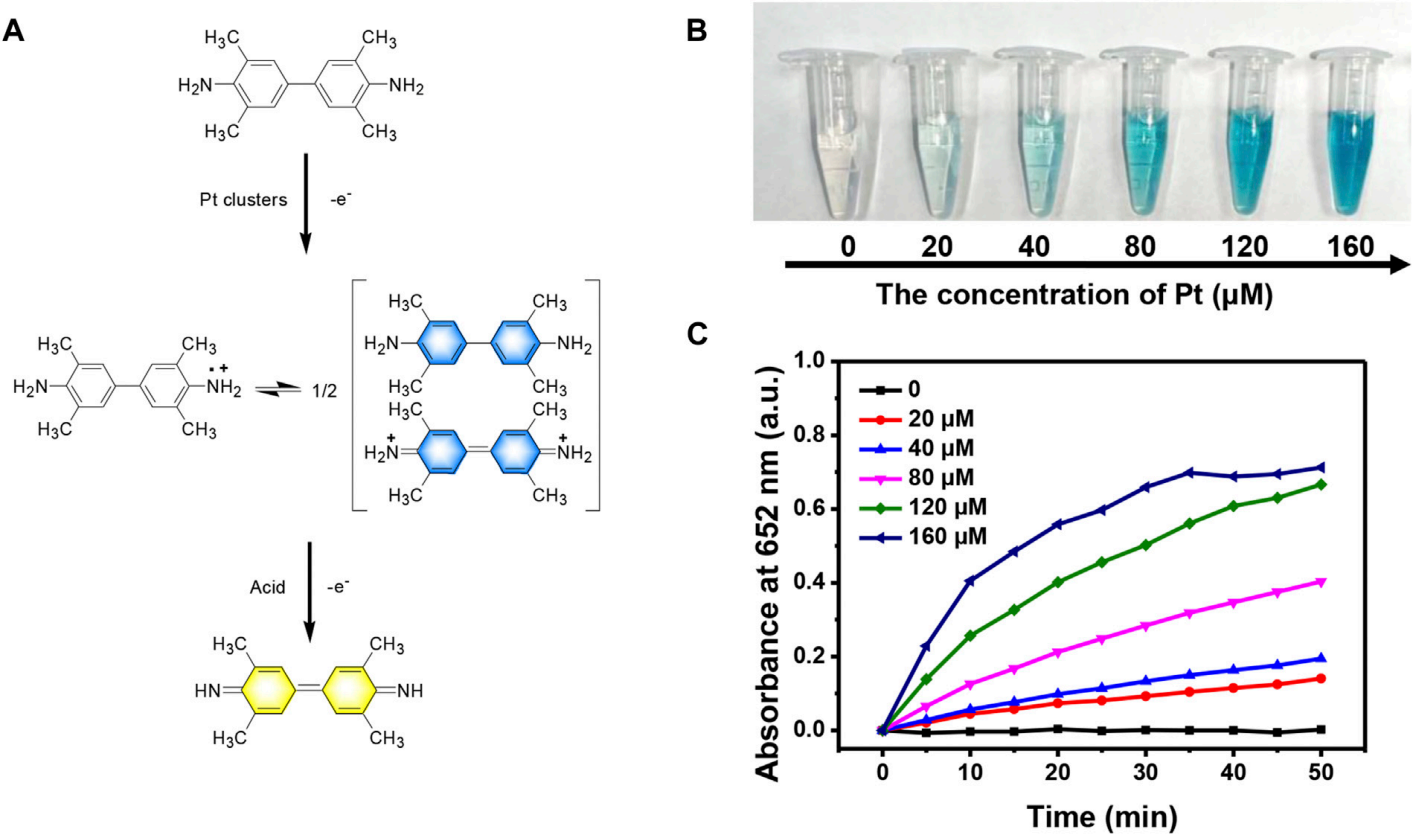
Peroxidase-like activity of Pt clusters. **(A)** Chemical reaction formula of TMB catalytic under Pt clusters; **(B)** Color changes of TMB at 5 min in the mixture containing 800 μM TMB and 100 μM H_2_O_2_ in the presence of Pt clusters with serial concentration. **(C)** Time-dependent absorbance change at 652 nm of the mixture containing 800 μM TMB and 100 μM H_2_O_2_ in the presence of Pt clusters in serial concentration.

We first evaluated the enzyme-like catalytic activity of Pt clusters by employing TMB and H_2_O_2_ as the substrates. In the presence of H_2_O_2_ and Pt clusters, the amino group of colorless TMB loses an electron to become a cationic radical and exists in the system in the form of a dimer charge-transfer complex. The dimer has the maximum absorption at 652 nm and appears blue. In an acidic environment, the complex loses another electron and is further oxidized into a yellow quinone conjugate monomer structure (Figure 3A). After the mixture of TMB and hydrogen peroxide was injected into Pt clusters of different concentrations, the colorless solution gradually turned blue. With the increase of Pt cluster concentration, the intensity of blue gradually increased (Figure 3B), and the maximum absorbance at 652 nm also gradually increased (Figure 3C). Without the introduction of Pt clusters, the color of TMB and the maximum absorbance did not change.

### 3.3 Studies of Pt clusters binding to the integrin in the cell membrane

**FIGURE 4:**
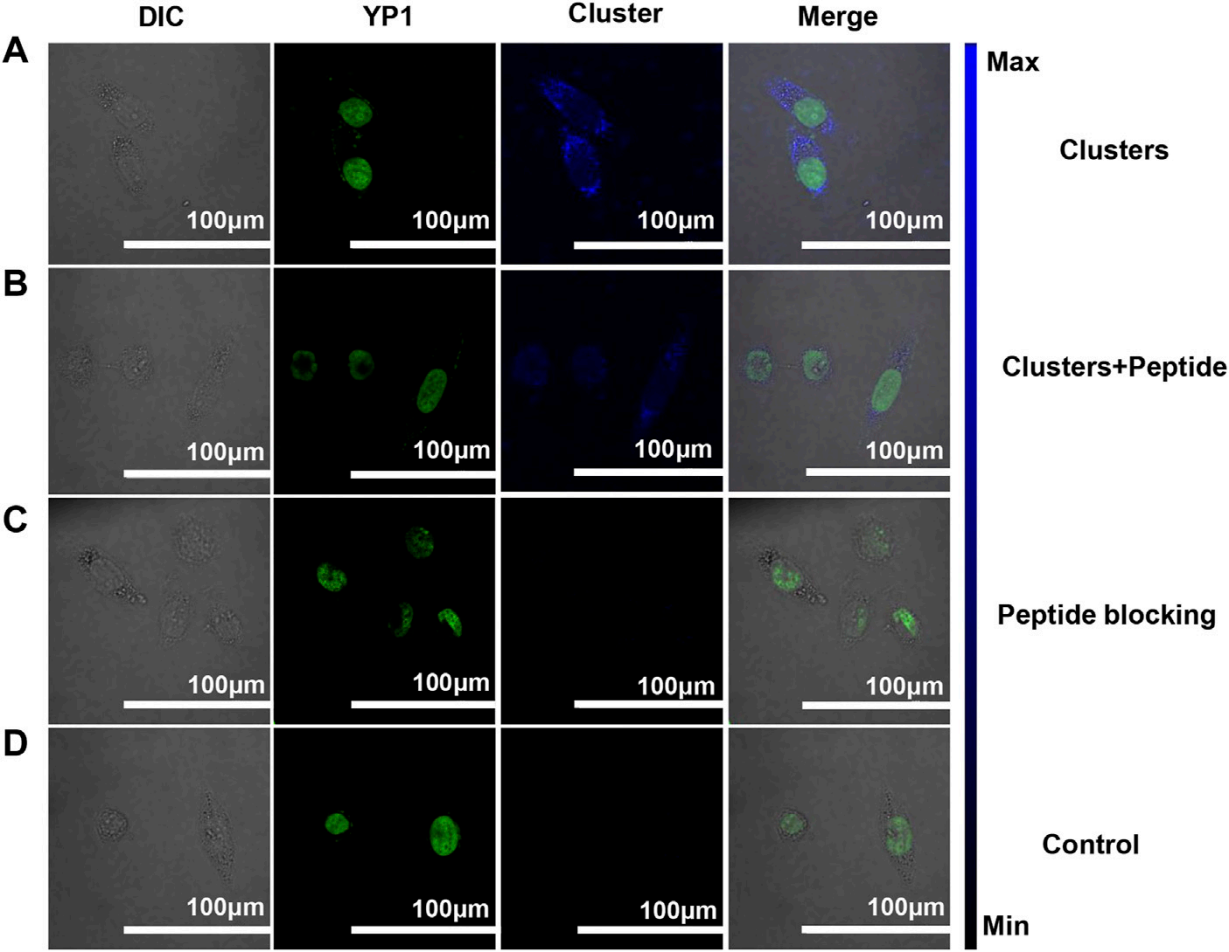
Pt clusters targeting *αvβ3* of SiHa cells were analyzed using the CLSM system. **(A)** SiHa cells were incubated with 120 μM Pt clusters for 1 h **(B)** 5 mM RGD peptides and 120 μM Pt clusters incubated for 1 h. **(C)** 5 mM RGD peptides were incubated for 1 h, followed by 120 μM Pt clusters incubation for 1 h. **(D)** Cells in medium without Pt clusters as a control.

Due to the targeting of RGD peptides, peptide-coated Pt clusters can bind with *αvβ3* expression in cells (Guo et al., 2014; Yuan et al., 2014; Ludwig et al., 2021; Pretze et al., 2021; Pang et al., 2023; Yin et al., 2023). Confocal laser scanning microscopy (CLSM) was used to verify the specificity target of Pt in SiHa cells. As expected, there was obvious blue fluorescence, as shown in Figure 4A, but no fluorescence in the control group (Figure 4D). This suggests Pt clusters are located on the cell membrane of SiHa cells. In addition, peptide competition and blocking experiments were performed to confirm the specific targeting of Pt clusters to *αvβ3*. When Pt clusters and free RGD peptides were added to cells, there was almost no blue fluorescence in the cells (Figure 4B), indicating that Pt clusters competed with free RGD peptides for the same binding site. When Pt clusters were introduced into cells after previously blocked by a 5 mM free RGD peptide, no blue fluorescence was observed in the cells (Figure 4C), suggesting that the integrin-binding site had been occupied by the free RGD peptide. The corresponding fluorescence intensity was analyzed by ImageJ software (Supplementary Figure S3). These results suggest that Pt clusters can specifically target *αvβ3* on SiHa cells.

### 3.4 Optimization of the Pt cluster concentration in cells

**FIGURE 5:**
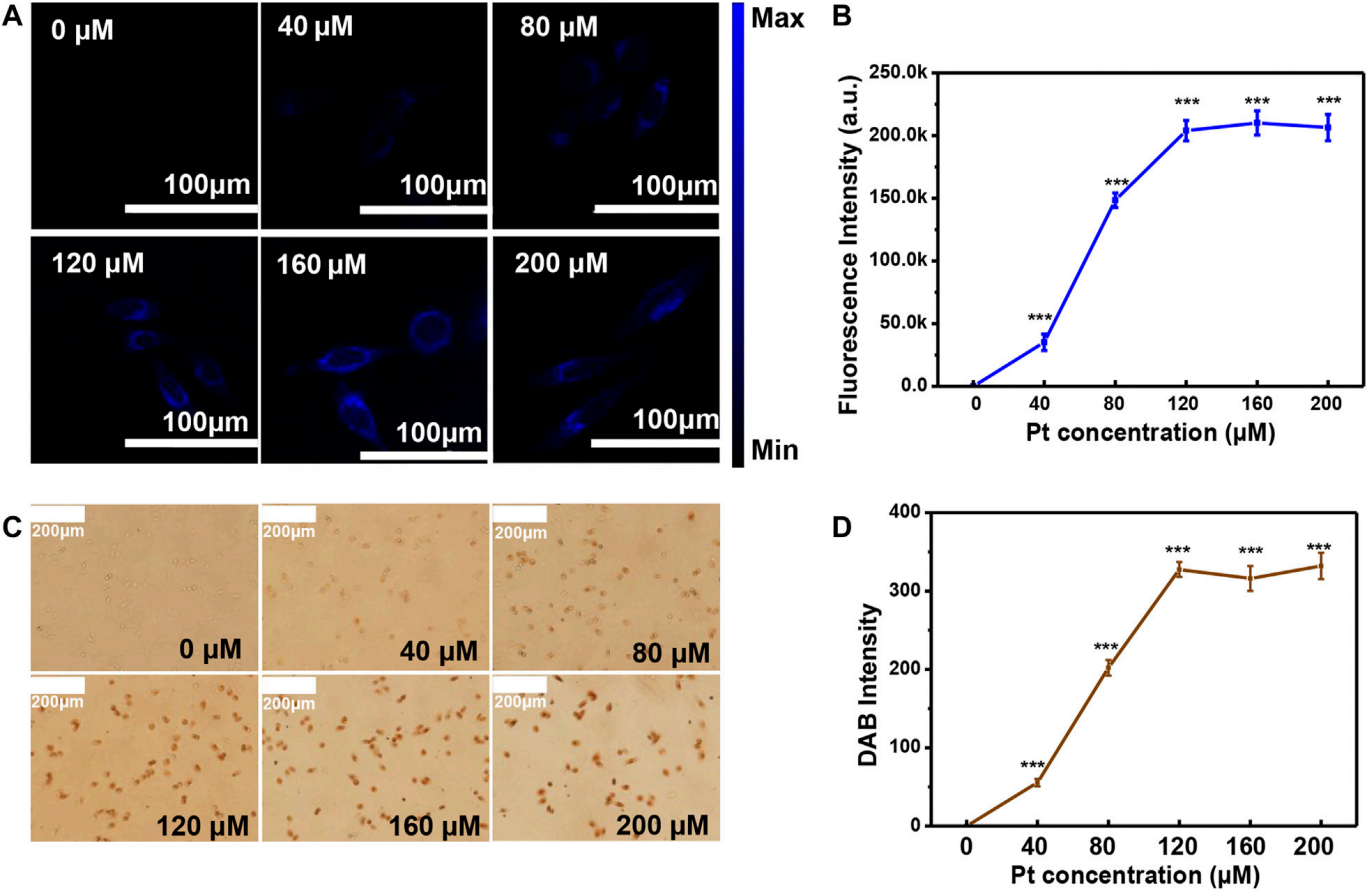
Optimization of the Pt cluster concentration in cell culture medium. **(A)** Confocal laser scanning microscopy (CLSM) images of SiHa cells exposed to different concentrations of Pt clusters for 60 min. **(B)** Corresponding fluorescence intensity analyzed by ImageJ software. **(C)** Images of the brown color cells when DAB are catalyzed by Pt clusters for 60 min. **(D)** Corresponding cell brown intensity analyzed by ImageJ software.

To detect *αvβ3* in the cell membrane more accurately, the optimal concentration of Pt cluster incubation with SiHa cells was studied. The fluorescence intensity of SiHa cells increased as the Pt cluster concentration increased from 0 to 120 μM (Figure 5A). The fluorescence intensity of SiHa cells stopped changing when the Pt cluster concentration reached 120 μM. In cell-cultured medium, when the Pt cluster concentration continued to increase over 120 μM, the fluorescence intensity did not increase significantly. This is consistent with the fluorescence intensity analyzed by ImageJ software (Figure 5B). After the cells were detected using the CLSM system, the DAB working solution was introduced into the cells previously incubated by the Pt cluster. Under the Pt cluster catalytic oxidation, DAB produced a brown sediment in the cells. The brown intensity of DAB increased as the Pt cluster concentration increased from 0 to 120 μM (Figure 5C). When the Pt cluster concentration reached 120 μM, the brown intensity of SiHa cells did not increase. The brown intensity on the cells was analyzed using ImageJ software. The result is consistent with naked eye observation under a normal light microscope. These results indicate that the saturation concentration of labeled *αvβ3* can be achieved when the concentration of Pt clusters is 120 μM in the cell medium.

### 3.5 Visual *αvβ3* protein expression level in three cell lines

**FIGURE 6:**
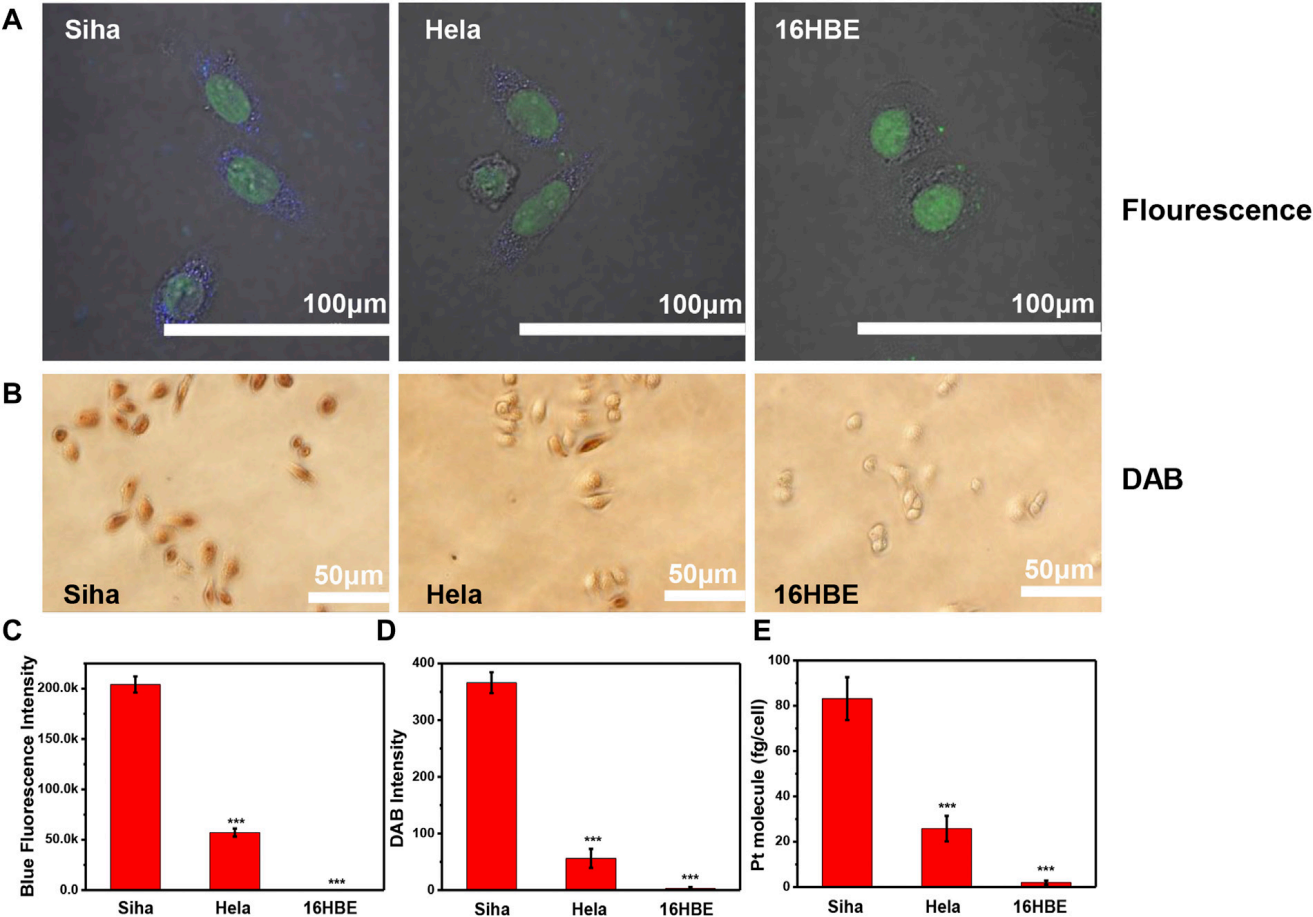
Detecting the expression level of *α*_v_ *β*_3_ among different cell lines. SiHa, HeLa, and 16HBE cells were exposed to 120 μM Pt clusters for 60 min **(A)** CLSM system imaging. **(B)** Images of the brown DAB dyed cells. **(C)** Corresponding fluorescence intensity analyzed by ImageJ software. **(D)** Corresponding brown intensity analyzed by ImageJ software. **(E)** Pt element content in the cell after cells were treated with Pt clusters.

To verify whether Pt clusters can easily and rapidly detect integrin expression on different cell lines, we selected SiHa, HeLa, and 16HBE cell lines with different integrin expressions as models. These cells were incubated with Pt clusters and observed using a fluorescence microscope. After this, the DAB working solution was added to the Pt cluster-labeled cells and observed using an optical microscope. As shown in Figures 6A,B, the brown sediment in SiHa cells was deeper than that in HeLa cells, indicating that SiHa cells expressed more integrins than HeLa cells. There was weak brown color on the surface of the 16HBE cell membrane, indicating that 16HBE cells expressed integrin *αvβ3* proteins at a low level. Brown intensity statistics also showed that SiHa cells had the highest expression of integrin *αvβ3* among the three cell lines (Figure 6C). The results were confirmed by changes in Pt cluster fluorescence intensity in the CLSM system (Figures 6A,B). The intensity of blue fluorescence in SiHa cells was stronger than that in HeLa cells, while it was almost undetectable in 16HBE cells. In addition, Pt clusters in SiHa and HeLa cells were quantified by ICP-MS, and the Pt mass in SiHa and HeLa cells was 83.16 fg/cell and 25.78 fg/cell, respectively. The near-absence of Pt in 16HBE cells further demonstrates that Pt clusters can differentiate integrin protein expression between different cell lines.

## 4 Conclusion

We constructed Pt clusters with specific molecular recognition and catalytic amplification signals for specific detection of *α*_v_ *β*_3_ expression levels in different tumor cell lines. The *α*_v_ *β*_3_ expression can be distinguished by the fluorescence properties of the Pt cluster. Integrin *αvβ3* expression can also be distinguished by ICP-MS *via* quantifying the mass of Pt. More importantly, the cell-binding Pt clusters can catalyze non-color DAB into brown-colored DAB *in situ*; thus, the integrin level in the cell can be directly seen by the naked eye. This method is simpler and does not require expensive detection equipment like a fluorescence microscope or mass spectra. Through our method, cells with different *αvβ3* expression levels can be quickly distinguished by observing the brown intensity under a common optical microscope. As *αvβ3* expression is positively correlated with the migration and invasiveness of tumor cells (Kwakwa and Sterling, 2017; Hight-Warburton and Parsons, 2019; Sales et al., 2019), this feature of Pt cluster catalytic properties provides a very easy approach to detect integrin in cells.

## Data Availability

The original contributions presented in the study are included in the article/Supplementary Material; further inquiries can be directed to the corresponding authors.
