# Supplementary material for: Observation of the protein expression level via naked eye: Pt clusters catalyze non-color molecules into brown-colored molecules in cells
Source: Front Chem. 2023 Feb 13;11:1145415. doi: 10.3389/fchem.2023.1145415 (PMC9969140; doi:10.3389/fchem.2023.1145415)
Supplement: Supplementary file 1 [file DataSheet1.docx]

Supplementary Material

**Seeing protein expression level via naked eyes: Pt clusters catalyze none color molecules into brown ones in cells**

Dongfang Xia^*^, Yong Zhang, Chunyu Zhang, Xiuxiu Yao, Yuhua Tang, Fuchao Wang **Correspondence:**

Hongzong Yin: [hzyin@sdau.edu.cn](mailto:hzyin@sdau.edu.cn)

Chao Xu: [xuc@sdau.edu.cn](mailto:xuc@sdau.edu.cn)

Xueyun Gao: [gaoxy@ihep.ac.cn](mailto:gaoxy@ihep.ac.cn)

# Supplementary Figures and Tables

## Supplementary Figures


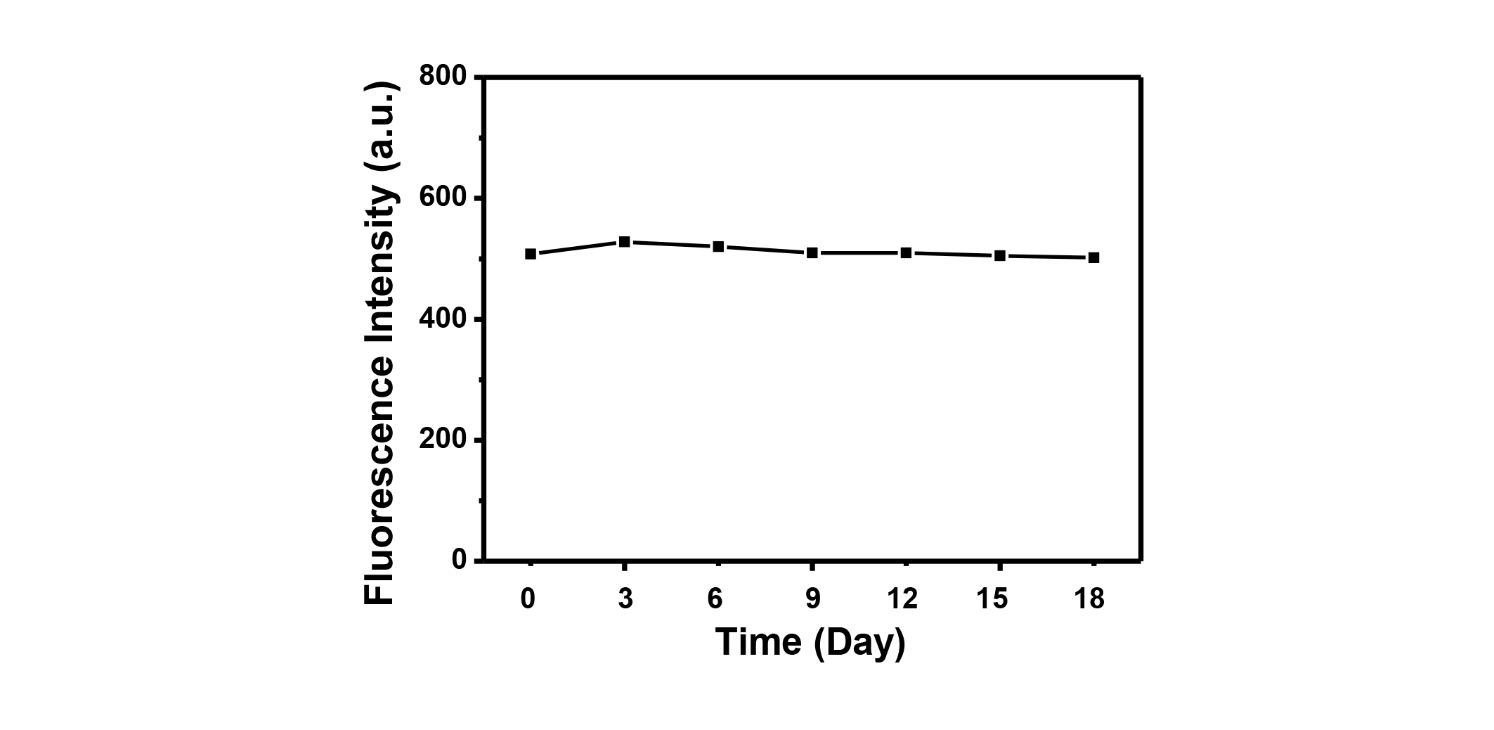
 **Supplementary Figure 1.** Stability of the Pt clusters in aqueous solution.


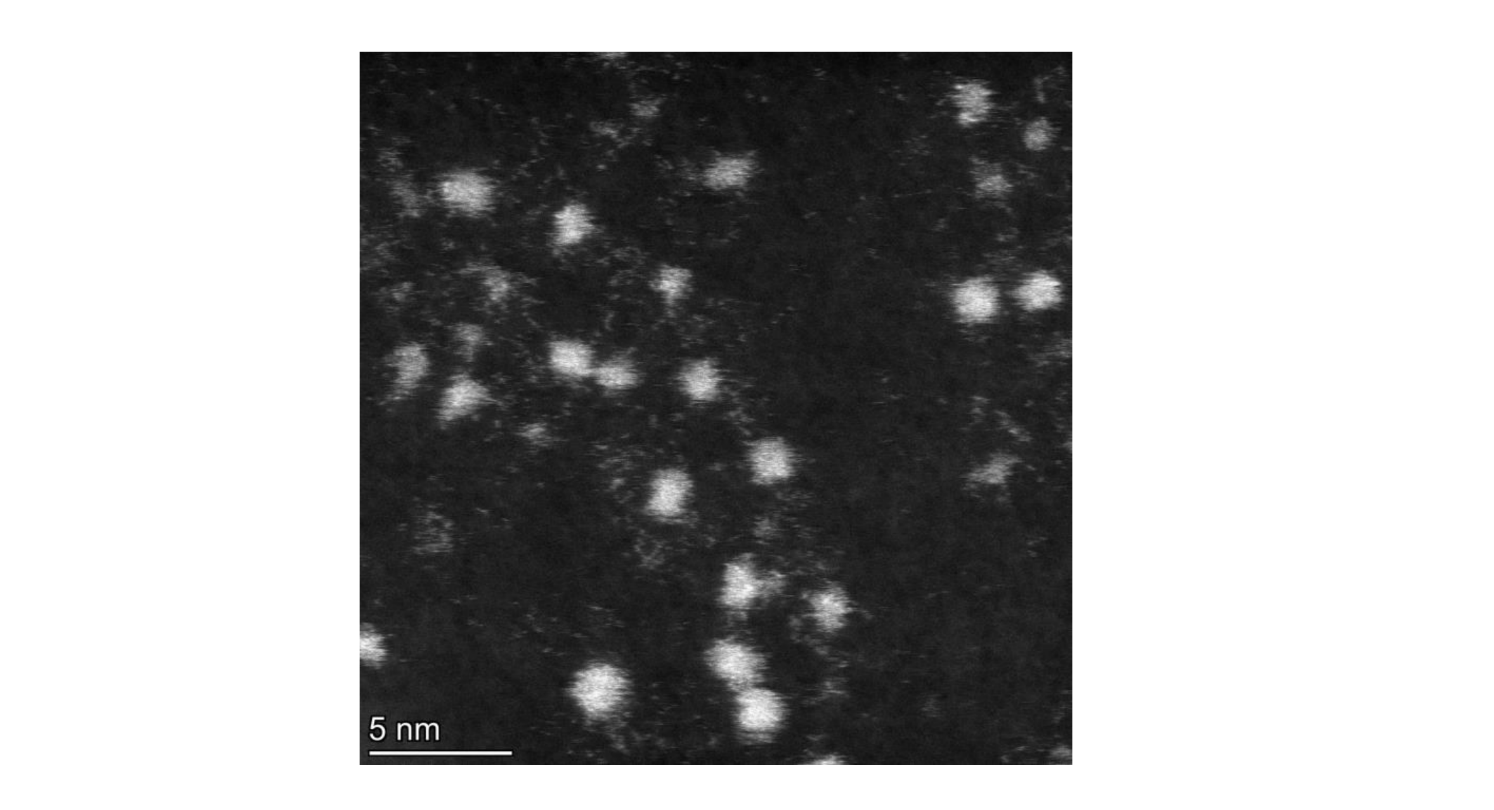


**Supplementary Figure 2.** TEM image of Pt clusters.

**
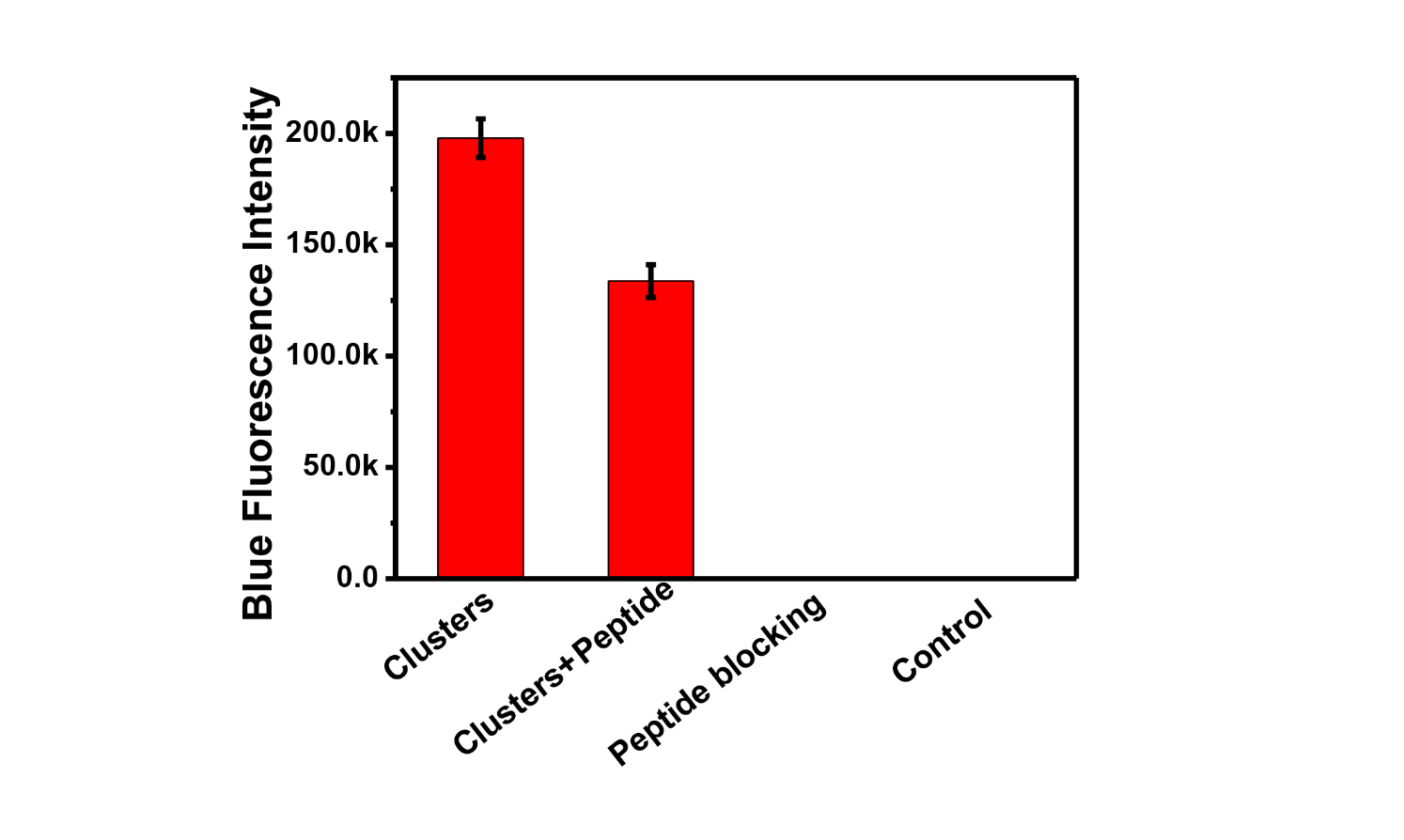
**

**Supplementary Figure 3.** The corresponding fluorescence intensity analyzed by imageJ software.
